# FTY720 elevates smooth muscle contraction of aorta and blood pressure in rats via ERK activation

**DOI:** 10.1002/prp2.308

**Published:** 2017-04-17

**Authors:** Zhen Zhao, Jinxin Wang, Zhijun Huo, Zhiyong Wang, Qibing Mei

**Affiliations:** ^1^ State Key Laboratory of New Drug & Pharmaceutical ProcessShanghai Institute of Pharmaceutical IndustryShanghai200437China

**Keywords:** ERK, PD98059, phosphorylation, S1P, stroke

## Abstract

Sphingosine 1‐phosphate (S1P) is an important signaling sphingolipid involved in the pathogenesis of various cardio cerebral vascular diseases such as ischemic stroke. In particular, the S1P mimetic FTY720 is protective for brain against ischemic conditions. However, whether and how FTY720 can modulate vascular tone and blood pressure remains to be determined. We showed that FTY720 (1 mg/kg) enhanced the contractile response of rat thoracic aortic rings induced by high potassium and phenylephrine, respectively. This enhancement involves the activation of extracellular signal‐regulated kinase (ERK) since ERK phosphorylation was also enhanced and application of PD98059 (10 *μ*mol/L), an inhibitor of ERK activation abrogated the aforementioned enhanced response by FTY720. In parallel, FTY720 (1 mg/kg) led to a modest elevation of blood pressure in rats, effects also being prevented by PD98059. In contrast, FTY720 decreased the high potassium‐induced contractile response in basilarartery preparations from rabbits, an effect blocked by PD98059. Together, FTY720‐induced an enhanced response of artery contractility in aorta and in arterial pressure involving ERK activation, with an attenuation in basilarartery contractility. This action property of FTY720 would be endowed with a potential of facilitating more blood flow perfusion to the brain and improving blood supply to the ischemic brain region and could be useful as an adjuvant in the treatment of ischemic stroke in the clinics.

**Abbreviations**
ERK: extracellular signal‐regulated kinase
PLC: phospholipase C
SPK: sphingosine kinase

## Introduction

Sphingosine 1‐phosphate (S1P), a bioactive sphingolipid metabolite acts as an intracellular messenger directly binding to its G protein‐coupled receptors (S1P receptor subtype 1–5) and regulates a number of cellular functions including cell proliferation and survival (Spiegel and Milstien 2003; Means and Brown 2009). Mounting evidence has shown that S1P can exert a protective role for heart and brain in response to ischemia‐reperfusion injury, either via direct action on cells or mobilization of neural progenitor cells (Kimura et al. 2008; Karliner 2013; Nagareddy et al. 2014; Abdel‐Latif et al. 2015). In addition, the S1P mimetic FTY720, a lipophilic immunomodulator has been approved for the treatment of relapsing‐remitting multiple sclerosis, which has also been found to be protective for heart and brain from ischemia‐reperfusion injury (Kraft et al. 2013; Rolland et al. 2013; Fu et al. 2014; Wang et al. 2014; Goltz et al. 2015).

FTY720, an S1P mimetic derived from myriocin, a component of the Chinese herb *Iscaria sinclarii* and approved for the treatment of relapsing‐remitting multiple sclerosis, has received much attention for its role in ischemic conditions (Karliner 2013; Kraft et al. 2013; Rolland et al. 2013; Fu et al. 2014; Wang et al. 2014). After endogenous phosphorylation by sphingosine kinase 2, FTY720 potently activates S1P_1_ receptors and binds less to S1P_3_ receptors (Karliner 2013). Application of FTY720 can enhance survival in isolated adult murine myocardiocytes, prevent ischemia‐reperfusion‐induced cardiac arrhythmias in an ex vivo rat heart model as well as reduce infarct size of murine heart subject to ischemia‐reperfusion (Karliner 2013; Fu et al. 2014; Wang et al. 2014). Furthermore, treatment with FTY720 is able to ameliorate ischemic stroke in animals or patients (Karliner 2013; Rolland et al. 2013; Fu et al. 2014).

Vasculature dysfunction has been linked to the pathogenesis of myocardial ischemia and ischemic stroke (Urbich and Dimmeler 2004; Winship et al. 2014; Lapi and Colantuoni 2015). In this regard, S1P induces vasorelaxation in rat coronary artery but vasoconstriction in canine or murine cerebral arteries, effects being mediated by S1P3 receptors. In aorta, S1P produces nitric oxide (NO)‐dependent vasorelaxation via activating the S1P3 receptors (Nofer et al. 2004; Roviezzo et al. 2006). These studies suggest a differential regulation of vascular contraction/relaxation function by S1P, in which the dilation of coronary artery by S1P may contribute to the restoration of blood supply in heart during myocardial ischemia and could explain, at least in part the protective role of S1P for heart in myocardial ischemia.

As aforementioned, FTY720 shows a protective role in ischemic stroke, in which condition the blood supply to the brain is largely decreased. However, to the best of our knowledge, whether the S1P mimetic FTY720 is able to regulate the vascular tone in cerebral arteries and peripheral arteries such as aorta and the consequent blood pressure remains to be clarified. In this study, we investigated the effects of pretreatment with FTY720 on vascular constriction/relaxation and blood pressure as well as the possible underlying mechanisms therein.

## Materials and Methods

### Animals

Male Sprague‐Dawley rats (230–250 g; provided by B&K Universal Group Limited, Shanghai, China) were used in this study. Animals were housed in a temperature‐ and light‐controlled room (12 h light‐dark cycle, lights on at 7:00 a.m), with free access to food and water. All experimental protocols were approved by the Institutional Ethics Committee and were in accordance with the guidelines of the International Association for the Study of Pain concerning the use of laboratory animals.

### Ex vivo tissue preparation

Animals were administered with a sphingosine‐1 phosphate mimetic, FTY720 (1 mg/kg, i.p.; from Selleck Chemicals, Houston, TX), and thoracic aorta were removed for ex vitro experiments 30 min later. Thoracic aortic rings were suspended within organ baths containing K‐H solution (all in mmol/L concentrations: NaCl 118, KCl 4.7, MgSO_4_ 1.2, KH_2_PO_4_ 1.2, NaHCO_3_ 25, CaCl_2_ 2.5, d‐glucose 10.6) bubbled with 95% O_2_ and 5% CO_2_. The temperature was set at 37°C. Following equilibrium of 30 min, the aortic rings were pre‐loaded with a resting tension. Isometric tension was transduced via a force transducer (JZJ01, Chengdu Instrumental Co., Chengdu, China), relayed and recorded real time via a computerized data acquisition system (RM6240BD, Chengdu Instrumental Co., Chengdu, China). For some preparations, endothelium was disrupted by a cotton rod and acetylcholine (1 *μ*mol/L) was used to test the integrity of endothelium. A <10% relaxation induced by acetylcholine (1 *μ*mol/L) against KCl (60 mmol/L) or phenylephrine (1 *μ*mol/L) was considered the removal/absence of endothelium. In addition, basilarartery preparations from rabbits were also used in this study. The tension was transduced and processed via an MPA2000 micro‐vascular system (Shanghai Alcott Biotech Co., Shanghai, China).

### Experimental protocols

Thoracic aortas were removed 30 min after an intraperitoneal injection of FTY720 (1 mg/kg) or saline (1 mL/kg). The dose of FTY720 was used based on previous publications (Kraft et al. 2013; Rolland et al. 2013; Fu et al. 2014; Wang et al. 2014; Goltz et al. 2015; Zhao et al. 2016). KCl and phenylephrine were employed as the contractile agents and were cumulatively administered. Several kinases including ERK, p38 and Akt were examined for their activation/phosphorylation by using western blotting. Our preliminary data showed the activation of ERK during the FTY720 treatment. An ERK activation inhibitor, PD98059 (from Cell Signaling Technology, Danvers, MA) was therefore added into the bath at least 15 min prior to the contractile agents, followed by the cumulative administration of KCl or phenylephrine.

Thirty minutes after the administration of FTY720, thoracic aortas were removed. And endothelium was removed or kept intact. KCl was cumulatively administered or a fixed dose of phenylephrine (10^−6^mol/L) was delivered.

For basilarartery preparations, rabbits were delivered with FTY720 (0.5 mg/kg) or saline (0.5 mL/kg) via auricular veins. Basilararteries were removed and prepared for ex vivo experiments 30 min post‐drug. KCl and phenylephrine were cumulatively administered.

### Western blotting

Animals were treated with FTY720 (1 mg/kg or 5 mg/kg). Thoracic aortas were removed 0.5 h post‐drug and homogenized on ice for 30 min in 100 *μ*L of RIPA lysis buffer containing 100 *μ*mol/L phenylmethanesulfonyl fluoride and 10*μ*g/mL aprotinin and leupepsin, and then centrifuged at 13,800 g for 25 min. The supernatants were collected and the protein concentrations were determined. Equal amount of protein (40 *μ*g/lane) was loaded and electrophoresed in a 12% sodium dodecyl sulfate‐polyacrylamide gel. After the electrophoresis, the proteins in the gels were transferred onto the PVDF membranes. The membrane was incubated overnight at 4°C with the primary antibodies including p‐ERK, p‐p38 and p‐Akt (all from Cell Signaling Technology, Danvers, MA) at a dilution of 1:1000. After rinsing with TBST for three times, the membrane was incubated with the HRP‐conjugated goat anti‐rabbit IgG for 1 h at room temperature. The protein bands were detected by using an ECL detection kit and then photographed with a FluorChem E imaging system (Protein Simple, Santa Clara, CA). For loading controls, membranes were incubated with a stripping buffer and reprobed with the *β*‐actin antibody (Santa Cruz Biotechnology, Santa Cruz, CA) at a dilution of 1:2000. The analysis of the grey level of each band was performed using the adobe photoshop 7.0 software. The grey level of each target band was normalized to its corresponding internal loading control. Relative grade was defined as the ratio of the normalized data of the treatment group relative to its control group.

### Blood pressure monitoring in anesthetized rats

Rats were anesthetized with intraperitoneal administration of chloral hydrate (400 mg/kg) and the right common carotid artery was exposed and cannulated via a transducer (TSD104A; BIOPAC Systems, Goleta, CA, USA). The arterial pressure signal was recorded real time via a computerized data acquisition system (MP150; BIOPAC Systems). The percentage changes of both systolic and dystolic blood pressure relative to baseline were used for statistical analyses.

### Statistical analyses

All data are expressed as mean ± standard error of the mean (SEM.). Unless otherwise indicated, statistical analyses were performed using a one‐way or two‐way ANOVA, wherever appropriate, followed by a post‐hoc student Newman‐Keuls test. A value of *P* < 0.05 was considered statistically significant.

## Results

### FTY720 pretreatment enhances the vascular contractile response induced by high potassium or phenylephrine in rat aortic rings

**Figure 1:**
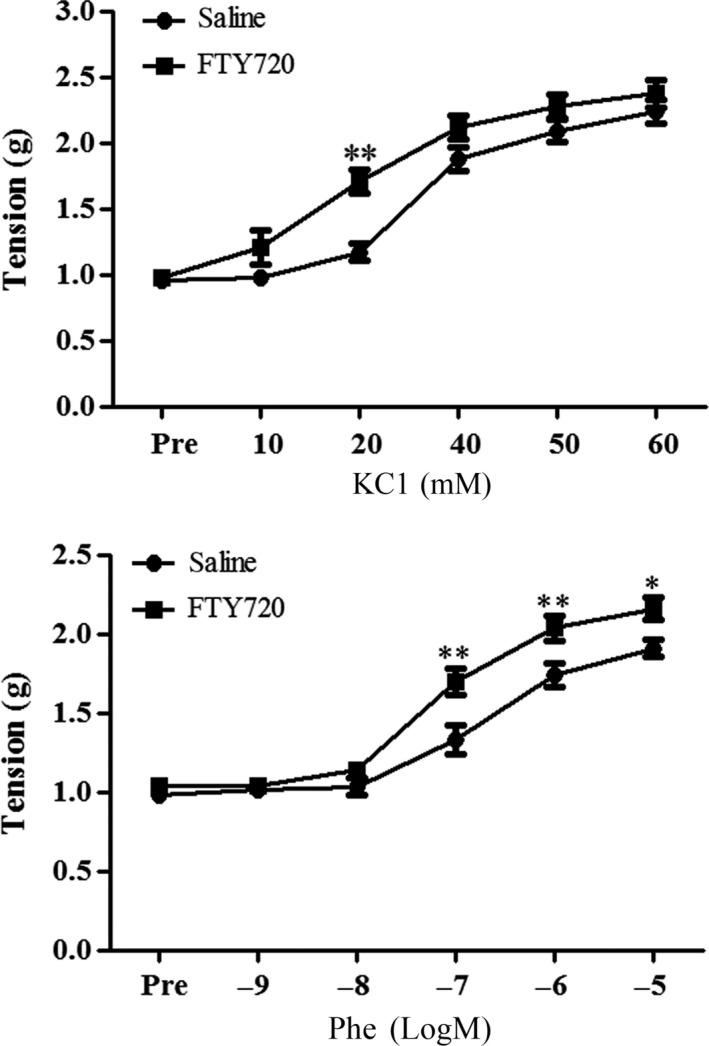
Effects of FTY720 on high potassium‐ or phenylephrine‐induced vasoconstriction in rat aorta. Saline or FTY720 (1 mg/kg) was intraperitoneally injected. Thoracic aortas were removed 30 min post‐injection and prepared for ex vivo experiments. Potassium chloride and phenylephrine (Phe) were used to induce vascular contraction. Data are expressed as mean ± SEM (*n* = 7–11/group). *P < 0.05 versus saline group; **P < 0.01 versus saline group.

As shown in Figure 1, a pretreatment with FTY720 (i.p., 1 mg/kg) enhanced the high potassium‐induced smooth muscular contractility (FTY720 group: 1.71 ± 0.09 versus Saline group: 1.17 ± 0.07 at 20 mmol/L of KCl; *P* < 0.01) as well as the phenylephrine (Phe)‐induced contraction of the rat aortic rings (FTY720 group: 1.70 ± 0.08 versus Saline group: 1.34 ± 0.09 at 10^−7^mol/L of Phe, *P* < 0.01; FTY720 group: 2.04 ± 0.08 versus Saline group: 1.74 ± 0.07 at 10^−6^mol/L of Phe, *P* < 0.01; FTY720 group: 2.16 ± 0.07 versus Saline group: 1.91 ± 0.05 at 10^−5^mol/L of Phe, *P* < 0.05). The dose‐response curve of vascular contraction induced by high potassium shifted left following FTY720 treatment (EC_50_ (mmol/L): 22.09 ± 1.09 in FTY720 group versus 32.95 ± 0.76 in saline group, *P* < 0.01; see Table 1). FTY720 treatment made a leftward shift of the Phe‐induced dose‐response curve of the aortic vascular contraction (pEC_50_: 7.08 ± 0.25 in FTY720 group versus 6.72 ± 0.26 in saline group; *P* < 0.05).

**Table 1:** Effects of various treatments on high potassium‐induced vascular contractility in rat aorta

Group		EC50 (mmol/L)	95% CI (mmol/L)
Saline	Saline	32.95 ± 0.76	31.43–34.46
FTY720	Saline	22.09 ± 1.09^a^	19.90–24.28
	DMSO	27.99 ± 1.76	24.41–31.57
	PD98059	35.23 ± 0.99^b^	33.22–37.24
	Endothelium	17.27 ± 0.32	16.61–17.94
	Endothelium denuded	17.07 ± 0.23	16.59–17.54

Data are expressed as mean ± SEM (*n* = 6–11/group). Saline or FTY720 (1 mg/kg) was intraperitoneally injected. Thoracic aortas were removed 30 min post‐injection and prepared for ex vivo experiments. Potassium chloride was used to induce vascular contraction.

a *P* < 0.01 versus Saline + saline group.

b *P* < 0.05 versus FTY720 + DMSO group.

### ERK activation is involved in FTY720‐induced enhancement of vascular contractility

**Figure 2:**
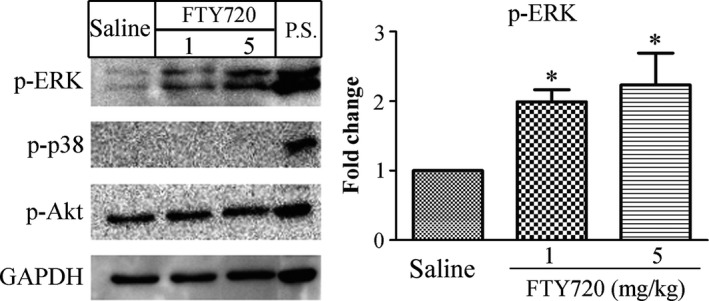
Kinase phosphorylation/activation in rat aorta following FTY720 treatment. Saline or FTY720 (1 or 5 mg/kg) was intraperitoneally injected. Thoracic aortas were removed 30 min post‐injection and homogenized in RIPA buffer for western blotting. Several kinases including ERK, p38 and Akt were detected for their phosphorylation. GAPDH was used as an internal control. Samples from rat brain were previously shown the expression of phosphorylated ERK, p38 and Akt, and thus used as the positive control (P.S.). Data are expressed as mean ± SEM (*n* = 3 rats/group). *P < 0.05 versus saline group.

**Figure 3:**
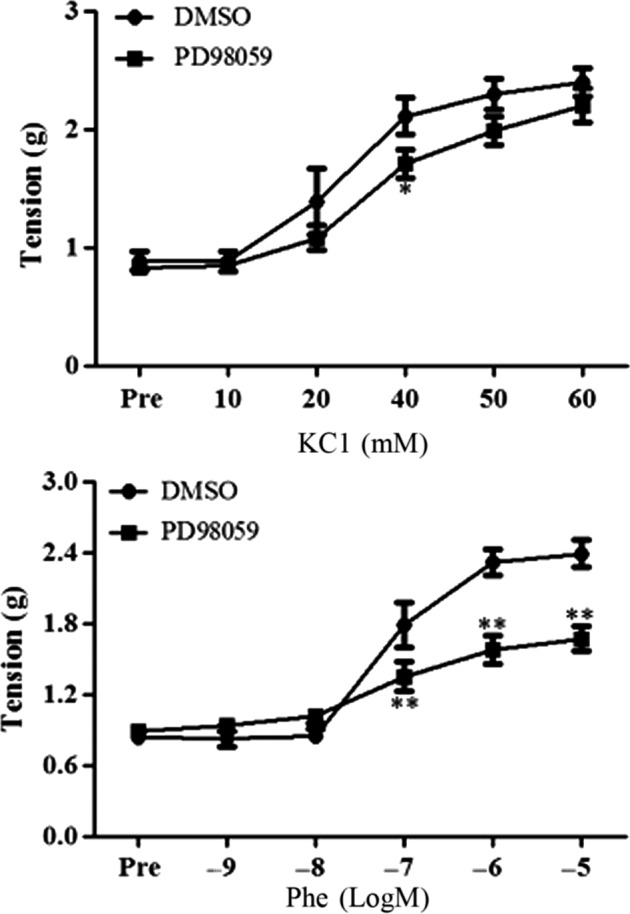
Effects of inhibition of ERK activation by PD98059 on high potassium‐ or phenylephrine‐induced vasoconstriction in rat aortas taken from FTY720‐treated animals. Animals were injected with FTY720 (1 mg/kg; i.p.) and thoracic aortas were removed 30 min post‐injection and prepared for ex vivo experiments. Aortic rings were pre‐incubated with PD98059 (10 μmol/L) or its vehicle DMSO for 10 min and then potassium chloride or phenylephrine (Phe) was added. Data are expressed as mean ± SEM (*n* = 5–8/group). *P < 0.05 versus DMSO group; **P < 0.01 versus DMSO group.

To investigate the possible signaling molecules underlying the enhanced vascular contractility by FTY720, several kinases including ERK, p38 and Akt were examined by western blotting for their phosphorylation/activation following FTY720 treatment. ERK phosphorylation (p‐ERK) was dose‐dependently increased by FTY720 while no change was found in the phosphorylated level of Akt (p‐Akt) or no phosphorylation of p38 (p‐p38) was observed by the FTY720 treatment (Fig. 2). We then asked whether inhibition of ERK activation could abrogate the FTY720‐induced enhancement of vascular contraction. As shown in Figure 3, pre‐incubation with PD98059 (10 *μ*M), an inhibitor of ERK activation, decreased the high potassium‐ or Phe‐induced vascular contractile responses in FTY720‐treated preparations (PD98059 group: 1.71 ± 0.12 versus DMSO group: 2.11 ± 0.16; *P* < 0.05 at 40 mM of KCl.) (PD98059 group: 1.70 ± 0.08 versus DMSO group: 1.34 ± 0.09 at 10^−7^mol/L of Phe, *P* < 0.01; PD98059 group: 1.58 ± 0.12 versus DMSO group: 2.32 ± 0.11 at 10^−6^mol/L of Phe, *P* < 0.01; PD98059 group: 1.67 ± 0.11 versus DMSO group: 2.39 ± 0.12 at 10^−5^mol/L of Phe, *P* < 0.01). The EC_50_ and pEC_50_ values were generalized in Tables 1 and 2 (EC_50_ of KCl (mmol/L):27.99 ± 1.76 in DMSO group versus 35.23 ± 0.99 in PD98059 group, *P* < 0.05; pEC_50_ of Phe: 7.16 ± 0.04 in DMSO group versus 6.78 ± 0.11 in PD98059 group; *P* < 0.05).

**Table 2:** Effects of various treatments on phenylephrine (Phe)‐induced vascular contractility in rat aorta

Group		pEC50(M)	95% CI
Saline	Saline	6.72 ± 0.26	6.20–7.24
FTY720	Saline	7.08 ± 0.25^a^	6.57–7.60
	DMSO	7.16 ± 0.04	7.07–7.24
	PD98059	6.78 ± 0.11^b^	6.56–7.00

Data are expressed as mean±SEM (*n* = 5–8/group). Saline or FTY720 (1 mg/kg) was intraperitoneally injected. Thoracic aortas were removed 30 min post‐injection and prepared for ex vivo experiments. Phenylephrine (Phe) was used to induce vascular contraction. The contraction potency was expressed as the negative logarithm of EC50 (pEC50).

a *P* < 0.05 versus Saline+saline group.

b *P* < 0.05 versus FTY720 + DMSO group.

### Possible effects of endothelium removal on the FTY720‐induced enhancement of vascular contractility

**Figure 4:**
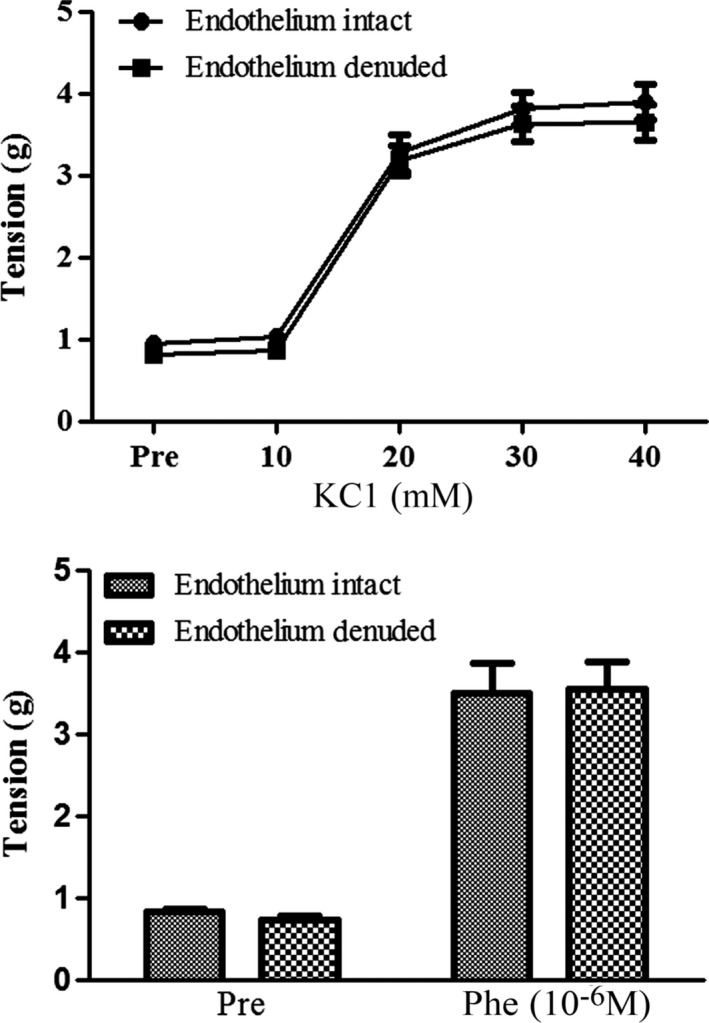
Effects of endothelium removal on high potassium‐ or phenylephrine‐induced vasoconstriction in rat aorta taken from FTY720‐treated rats. Animals were injected with FTY720 (1 mg/kg; i.p.) and thoracic aortas were removed 30 min post‐injection and prepared for ex vivo experiments. Aortic rings were rubbed by a wooden rod to remove endothelium. Potassium chloride and phenylephrine (Phe) was used to induce vascular contraction. Data are expressed as mean ± SEM (*n* = 6/group).

It is well known that endothelial cells may release nitric oxide and thus influence vascular contraction or relaxation. To test whether this factor could be involved in the FTY720‐induced vascular contractile enhancement, we denuded the endothelium and probed the consequences of vascular responses in FTY720‐treated preparations. As shown in Figure 4 and Table 1, endothelium removal seemed to have no significant influences on the high potassium‐ or Phe‐induced vascular contractility (Phe: 10^−6^mol/L) in rat aortic rings taken from FTY720‐pretreated animals.

### Possible effects of FTY720 on vascular contractility of basilarartery induced by high potassium

**Figure 5:**
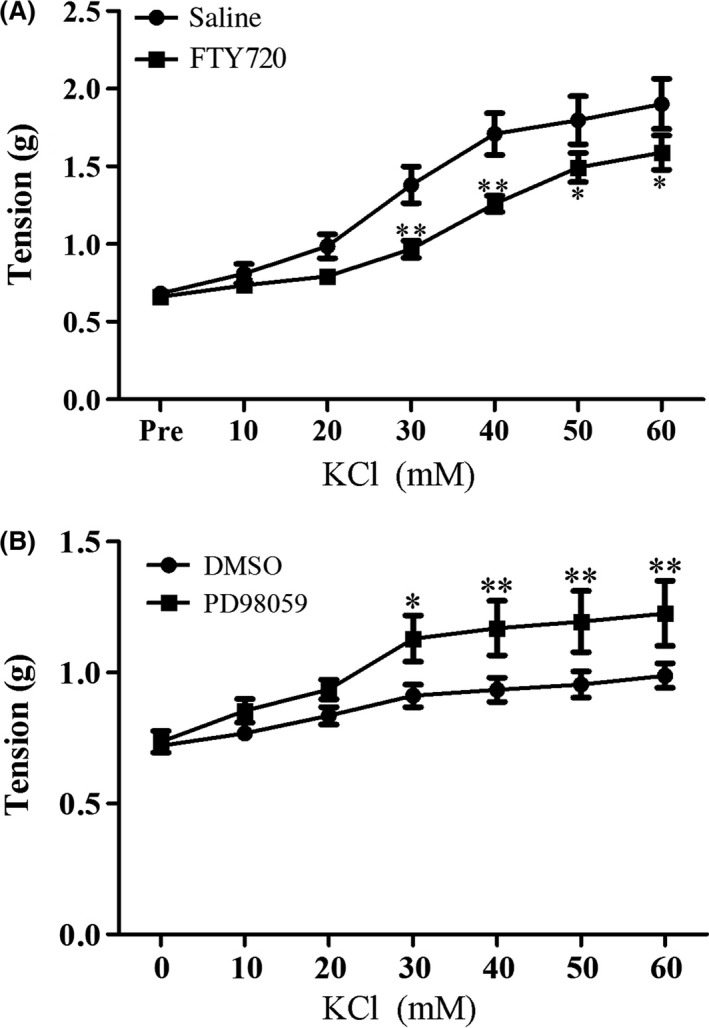
Effects of various treatments on high potassium‐induced vasoconstriction in rabbit basilarartery. (A) Saline or FTY720 (1 mg/kg) was intraperitoneally injected. Basilararteries were removed 30 min post‐injection and prepared for ex vivo experiments. Potassium chloride was used to induce vascular contraction. Data are expressed as mean ± SEM (*n* = 6–8/group). *P < 0.05 versus saline group; **P < 0.01 versus saline group. (B) Animals were injected with FTY720 (1 mg/kg; i.p.) and thoracic aortas were removed 30 min post‐injection and prepared for ex vivo experiments. Basilarartery preparations were pre‐incubated with PD98059 (10 *μ*mol/L) or its vehicle DMSO for 10 min and then KCl was added. Data are expressed as mean ± SEM (*n* = 7–8/group). *P < 0.05 versus saline group; **P < 0.01 versus saline group.

Besides aorta, basilarartery was also prepared ex vivo to examine the possible effects on the vascular contraction by interruption of S1P signaling using FTY720. We first used phenylephrine as the contractive agent and observed a weak contractile response of basilarartery preparations (data not shown), effects different from KCl‐induced contraction. As shown in Figure 5, pretreatment with FTY720 led to a decrease of high potassium‐induced vascular contraction in rabbit basilarartery preparations (FTY720 group: 0.97 ± 0.05 versus Saline group: 1.38 ± 0.12 at 30 mmol/L of KCl, *P* < 0.01; FTY720 group: 1.26 ± 0.05 versus Saline group: 1.71 ± 0.13 at 40 mmol/L of KCl, *P* < 0.01; FTY720 group: 1.49 ± 0.09 versus Saline group: 1.80 ± 0.15 at 50 mmol/L< of KCl, *P* < 0.05; FTY720 group: 1.59 ± 0.11 versus Saline group: 1.90 ± 0.16 at 60 mmol/L of KCl, *P* < 0.05) (See Table 3. EC_50_(mmol/L): 26.55 ± 1.45 in saline group versus 34.65 ± 1.83 in FTY720 group, *P* < 0.05). Interestingly, for those basilarartery preparations taken from FTY720‐treated animals, inhibition of ERK activation by PD98059 (10 *μ*mol/L) abrogated FTY720‐induced decrease of high potassium‐associated vascular contraction (EC_50_(mmol/L): 32.46 ± 2.27 in DMSO group versus 18.78 ± 1.36 in PD98059 group, *P* < 0.05).

**Table 3:** Effects of various treatments on high potassium‐induced vascular contractility in rabbit basilarartery

Group		EC50 (mmol/L)	95% CI (mmol/L)
Saline	Saline	26.66 ± 1.45	23.75–29.57
FTY720	Saline	34.65 ± 1.83^a^	30.95–38.36
	DMSO	32.46 ± 2.27	27.88–37.04
	PD98059	18.78 ± 1.36^b^	16.03–21.53

Data are expressed as mean ± SEM (*n* = 6–8/group). Saline or FTY720 (0.5 mg/kg) was intraperitoneally injected. Basilararteries were removed 30 min post‐injection and prepared for ex vivo experiments. Potassium chloride was used to induce vascular contraction.

a *P* < 0.01 versus Saline+saline group.

b *P* < 0.05 versus FTY720 + DMSO group.

### Possible effects of FTY720 on blood pressure in anesthetized rats

**Figure 6:**
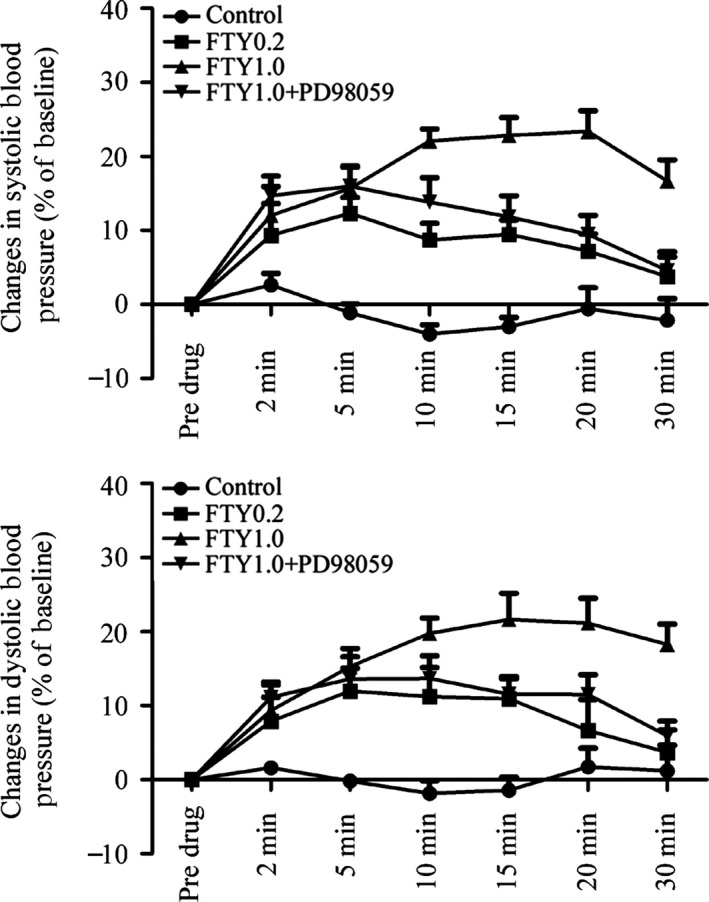
Changes of arterial pressure following FTY720 treatment alone and in combination with PD98059. FTY720 (0.2 mg/kg, *n* = 7, FTY0.2; 1.0 mg/kg, *n* = 6, FTY1.0) or saline (0.9%, 1.0 mL/kg, *n* = 9, Control) was intraperitoneally injected in anesthetized rats. The ERK activation inhibitor, PD98059 (40 *μ*g/kg, *n* = 7; i.p.) was used to reveal possible ERK involvement and injected 10 min prior to FTY720 treatment. Data are expressed as mean ± SEM.

We further monitored blood pressure changes following FTY720 treatment (0.2 and 1.0 mg/kg; i.p.) in anesthetized rats. As shown in Figure 6, FTY720 evoked dose‐ and time‐dependent increases in systolic and dystolic blood pressure, effects being abrogated by the ERK activation inhibitor, PD98059 (40 *μ*g/kg; i.p.).

## Discussion

In ischemic stroke, the occlusion of middle cerebral artery due to thromboembolism leads to a decrease of blood supply to brain. The augmentation of contraction of peripheral large artery (aorta) and blood pressure while attenuation of contraction of cerebral arteries (basilar artery) would be beneficial to enhance the blood flow perfusion of ischemic brain region, thereby improving the ischemic state and promoting the neuronal survival. In this scenario, we have examined the effects of pretreatment with FTY720 on vascular tone and arterial pressure and identified that FTY720 enhances the vasoconstriction in aorta while attenuates the vasoconstriction in basilarartery. This differential regulation seems to be mediated by ERK activation since the phosphorylation (activation) of ERK is increased following treatment with FTY720 and application of ERK activation inhibitor PD98059 can abrogate the FTY720‐induced responses. Meanwhile, FTY720 also induces an ERK‐dependent increase in blood pressure.

Of the five G protein‐coupled S1P receptors (S1P_1‐5_), S1P_1_ receptors are only coupled to G_*α*i_, leading to the inhibition of adenylyl cyclase with S1P_2_ and S1P_3_ receptors coupled to G_*α*i_, G_*α*q_ and G_*α*12/13_ and S1P4 and S1P5 receptors coupled chiefly to G_*α*i_ and G_*α*12/13_ (Coussin et al. 2002). When detecting their distribution, only S1P_1‐3_ receptors are mostly enriched in cardiovascular tissues (including thoracic aortas) and cerebral arteries (Coussin et al. 2002; Schuchardt et al. 2011; Waeber and Walther 2014). Further studies have shown that modulation of S1P receptors (predominantly S1P_3_ receptor subtype) by S1P can regulate vascular tone and organ perfusion in heart and brain (Schuchardt et al. 2011). In detail, S1P can induce vasoconstriction in resistance arteries such as basilararteries while vasorelaxation in both aortas and coronary arteries, with S1P3 receptors involved in the dual effects (Mair et al. 2010; Murakami et al. 2010; Salomone et al. 2010; Schuchardt et al. 2011). Moreover, S1P‐induced vasorelaxation of aorta is endothelial NO‐dependent (Nofer et al. 2004; Roviezzo et al. 2006). In contrast to S1P‐related vascular relaxation in aorta, pretreatment with FTY720 led to an enhancement of vascular contraction induced by high potassium or phenylephrine, an effect independent of endothelium. Considering that FTY720 has been endowed with a property of activation of S1P_3_ receptors and activation of this receptor subtype has been linked to the increase in intracellular Ca^2+^ concentration in cultured smooth muscle cells, it is thus tempting to speculate that the regulation of vascular tone in aorta by pretreatment with FTY720 could be ascribed to its action on S1P_3_ receptors located in aortic smooth muscle cells (Karliner 2013; Fujii et al. 2014). One may argue that activation of S1P_1_ receptors could also mediate FTY720‐related modulation of vascular contraction in aortic rings since FTY720 does bind potently to this S1P_1_ receptor subtype (Karliner 2013). However, mounting evidence has demonstrated that there are relatively lower levels of S1P_1_ receptor expression than the other receptor subtypes on vascular smooth cells and activation of S1P_1_ receptors only slightly increases intracellular calcium through G_*βγ*._ S1P_3_ receptors are the main factor leading to the increase in intracellular calcium through the activation of phospholipase C (PLC) since deletion of this receptor subtype led to the marked inhibition of PLC activation (Ishii et al. 2002; Alewijnse et al. 2004; Waeber et al. 2004; Watterson et al. 2005; Peters and Alewijnse 2007; Murakami et al. 2010).

On the other hand, in contrast to its enhancement of vascoconstriction in aorta, pretreatment with FTY720 attenuated the high potassium‐induced vascular contraction in our basilarartery preparations. Consistent with our observation, Salomone and colleagues proposed that FTY720 decreased vasoconstriction in basilararteries induced by other vasoconstrictive agents including 5‐HT (5‐hydroxytryptamine) and S1P (Salomone et al. 2010). The FTY720‐induced related effects in basilarartery have been ascribed to its inhibition of sphingosine kinase (SPK), the key of S1P‐synthesizing enzyme. Indeed, SPK activity can be stimulated by agonists of various GPCRs as well as by depolarization‐induced Ca^2+^ entry (i.e., application of high potassium) and its activation can lead to activate a RhoA/Rho kinase pathway, thereby up‐regulating calcium levels and phosphorylating myosin light chain in vascular smooth cells and inducing contraction (Alemany et al. 2001, 2007; Bolz et al. 2003; Somlyo and Somlyo 2003; Salomone et al. 2010). In this scenario, it is reasonable to speculate that SPK inhibition could lead to a decrease of vasoconstriction. Alternatively, S1P_1_ and S1P_3_ receptors are mostly abundant in endothelial cells (ECs), involved in a variety of processes including cell migration, proliferation and regulation of endothelial barrier integrity (Peters and Alewijnse 2007). The predominant modulation of S1P_3_ receptors has been reported to evoke an increase in intracellular Ca^2+^ and the activation of eNOS, which results in NO release and vasorelaxation (Nofer et al. 2004; Roviezzo et al. 2006). In this regard, we infer that the modulation of S1P_3_ receptors located on basilarartery ECs by pretreatment with FTY720 underlies the FTY720‐related decrease of vasoconstriction by high potassium in basilararteries.

Various kinases such as ERK, p38, Akt, RhoA/Rho have been linked to the downstream events following S1P receptor activation. For instance, activation of S1P_3_ receptors leads to the activation of Akt and eNOS (Nofer et al. 2004; Roviezzo et al. 2006). S1P‐induced ERK activation has been observed in aorta as well as in basilararteries (Coussin et al. 2002). In our study, we observed only ERK activation/phosphorylation following FTY720 treatment. No p38 phosphorylation was detected and Akt phosphorylation was of no change. These characteristics of FTY720 may explain the differential effects of FTY720 *per se* and S1P in the contractile responses. In addition, application of ERK activation inhibitor PD98059 abrogated FTY720‐related effects in the contractile responses in both aorta and basilararteries, indicating ERK involvement.

In conclusion, contrary to the S1P‐induced effects in aorta and basilarartery, FTY720 induced an enhanced response of artery contractility in aorta and arterial pressure involving ERK activation, with an attenuation in basilarartery contractility. This action property of FTY720 would be endowed with a potential of facilitating more blood flow perfusion to the brain and improving blood supply to the ischemic brain region and could be useful as an adjuvant in the treatment of ischemic stroke in the clinics.

## Disclosures

Authors have declared that they have no conflict of interest.
